# New Insights into the Cystine-Sulfite Reaction

**DOI:** 10.3390/molecules24132377

**Published:** 2019-06-27

**Authors:** Matteo Zecchini, Robert Lucas, Adam Le Gresley

**Affiliations:** 1 Life Sciences, Pharmacy and Chemistry, Kingston University, Surrey, KT1 2EE, Kingston-upon-Thames, UK; 2 GlaxoSmithKline, Consumer Healthcare, Weybridge, KT13 0DE, UK

**Keywords:** cysteine, sulfite, cysteine-S-sulfate, photochemistry, mechanism

## Abstract

The mechanism by which cysteine-S-sulfate is formed from the reaction of sulfite with cystine in the absence of a dedicated oxidizing agent is investigated using high-resolution NMR. Changes to reactant ratio, pH, UV light exposure and temperature were evaluated to determine the most effective conditions to achieve the maximum yield of cysteine-S-sulfate without recourse to conventional oxidizing reagents. Herein evidence is provided for both nucleophilic and radical mechanisms, by which cysteine-S-sulfate can be generated with yields of up to 96%.

## 1. Introduction

Cysteine-S-sulfate (SSC) is produced by the reaction of inorganic sulfite and cystine and is a very potent N-methyl-D-aspartate-receptor (NMDA-R) agonist. Electrophysiological studies have shown that SSC displays depolarizing properties similar to glutamate. Patients affected with either Molybdenum cofactor deficiency (MOCOD, an autosomal recessive disease that leads to a combined deficiency of the enzymes sulfite oxidase, an enzyme that catalyzes the conversion of sulfite to inorganic sulfate, xanthine dehydrogenase and aldehyde oxidase) or isolated sulfite oxidase deficiency (ISOD, an extremely rare autosomal recessive disorder with identical clinical manifestations to MOCOD) excrete elevated levels of SSC. This rare disorder is associated with brain damage (seizures, spastic quadriplegia, and cerebral atrophy), mental retardation, dislocated ocular lenses, blindness, and excretion in the urine of abnormally large amounts of SSC, sulfite, and thiosulfate but no inorganic sulfate [1].

SSC and thiosulfate have also been suggested as intermediates in the conversion of inorganic sulfate to organic sulfur compounds by molds [2,3,4,5]. This suggestion is based on growth studies with various mutant strains of *Neurosptn-a* and Aspergillus and on the observation that a mutant strain of *Aspergilh* will accumulate trace amounts of cysteine-S-sulfate intracellularly.

A further application of SSC is in the industrial fed-batch cultivation of mammalian cells, used for the production of therapeutic proteins such as monoclonal antibodies. Besides medium ensuring initial growth, feeding is necessary to improve growth, viability, and antibody production. Established processes include a slight acidic main feed and a separate alkaline feed containing l-tyrosine and l-cysteine. Since L-cysteine is not stable at neutral pH, SSC, has been used in neutral pH feeds. In small scale fed-batch processes, the SSC process yielded a comparable maximum viable cell density, prolonged viability, and increased titer compared to the two-feed system. Bioreactor experiments confirmed the increase in specific productivity. In-depth characterization of the monoclonal antibody indicated no change in the glycosylation or charge variant pattern whereas peptide mapping experiments were not able to detect any integration of the modified amino acid in the sequence of the monoclonal antibody. Finally, the mechanism of action of SSC was investigated, and results pointed out the anti-oxidative potential of the molecule, mediated through an increase in superoxide dismutase enzyme levels and in the total intracellular glutathione pool. It has also been reported that this increase in specific productivity obtained in the SSC process results from the anti-oxidative properties of the molecule.

**Scheme 1:**
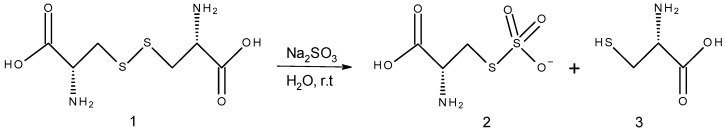
Reported reaction as per Clark et al. [7].

Synthesis of SSC has previously been reported but mostly relies on a dedicated oxidizing agent, aggressive solvents such as ammonia [6] or provide less than satisfactory yields. The mechanism for the reaction of sulfite with cystine to yield SSC was reported in the 1930s, and all subsequent papers are based on this understanding of the mechanism [7,8] (Scheme 1):

Subsequent investigations [8] indicated that the deliberate oxidation of the resultant cysteine by-product was necessary in order to facilitate the generation of high yields of SSC. For the reported investigation, NMR was chosen as the best technique for monitoring the reaction as it enables the characterization and quantitation of components in situ. It also enables the application of more complex techniques for mixture characterization, e.g., DOSY, without the need for complicated purification techniques.

**Figure 1:**
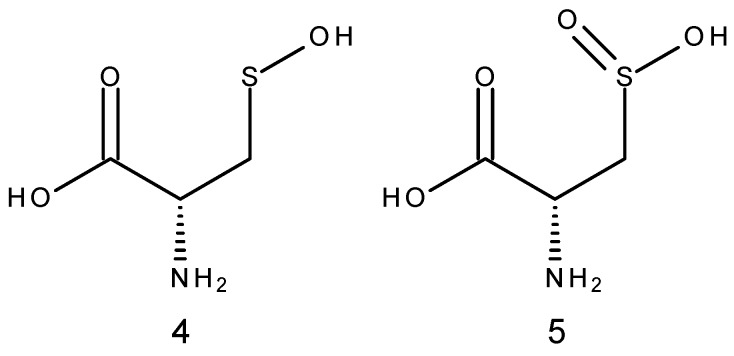
Cysteine oxidation products sulfenic acid 4 and sulfinic acid 5.

Herein, we report the rapid, spontaneous formation of a sulfenic acid oxidation product 4 which readily forms SSC on standing (Figure 1). The effects of cystine/sulfite concentrations, pH, Acid cycling and UV irradiation are discussed in the context of the distribution of possible products and the proposed mechanism.

## 2. Results

### 2.1. Intermediates and Mechanism Postulation

#### 2.1.1. Initial NMR Observations

**Figure 2:**
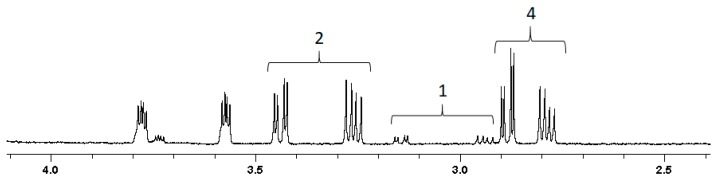
^1^H-NMR spectra extract (4.2–2.2 ppm) for cystine:sulfite (1:10) with a [SO_3_^2−^] = 0.1 M, after 20 min at pH 8–9 in the region.

Initial experiments involved a cystine:sulfite mixture (1:10) in distilled water at room temperature. The reaction was monitored by ^1^H-NMR after 20 min as reported below (Figure 2).

**Figure 3:**
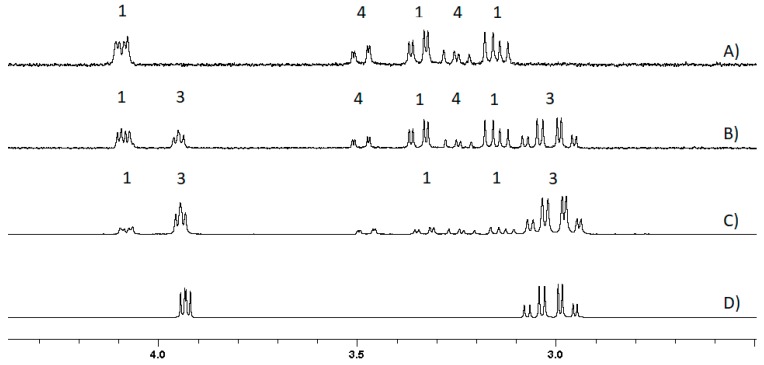
Stacked plots of ^1^H-NMR from cysteine oxidation reaction with 0.1 eq of H_2_O_2_ after (**A**) overnight, (**B**) 1 h, (**C**) 10 min and comparison with the (**D**) starting material.

NMR analysis of cysteine **3** at the same pH (8–9), shows the observed signals for the CH_2_ group of the anticipated cysteine in the reaction mixture are at lower field than cysteine alone and with a more defined splitting pattern (Supplementary Materials Figure S1) and hence cannot actually be cysteine **3**. SSC is also formed in larger quantities over time in the absence of any specific oxidising agent other than residual dissolved oxygen, supporting the suggestion of an oxidised species to react with further quantities of sulfite. To determine if this sulfenic acid intermediate **4** could be generated using a standard oxdising agent, cysteine was dissolved in H_2_O and H_2_O_2_. Figure 3 indicates the immediate formation of the same cysteine splitting pattern and the increase in signal intensity for both cystine and what is believed to be sulfinic acid **5**, further downfield.

**Figure 4:**
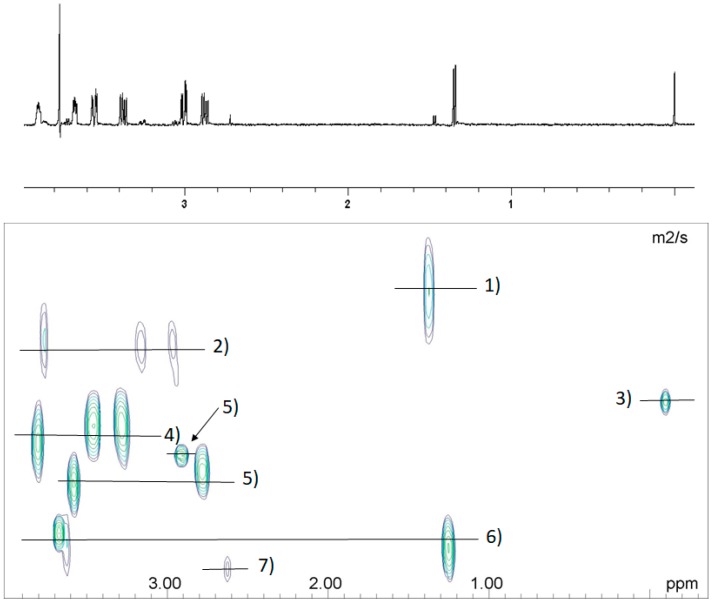
DOSY data based on their diffusion coefficient and peak assignment, where (**1**) and (**7**) are unassigned peaks, (**2**) cystine 1, (**3**) TSP, (**4**) sulfocysteine 2, (**5**) sulfenic acid 4 and (**6**) l-Alanine methyl ester.

The unusual NMR signals for the anticipated cysteine led to the use of MS techniques to identify the species or confirm that it was cysteine. Unfortunately, the individual components could not be isolated using prep TLC, before decomposing or converting. This led to the use of an in-situ technique —DOSY-NMR. This is a well-established technique to probe the relative diffusions of different molecules and has been used to determine approximate molecular weights for molecules in mixtures [9]. The purpose of characterizing the mixture using such a technique was to gain more evidence that our intermediate was a species heavier than cysteine (Figure 4). The expected MW of the cysteine sulfenic acid **4** is 137 g mol^−1^, while cysteine **3** has an MW of 121 g mol^−1^. The analysis was carried out as per the materials and methods, using two internal standards, TSP and l-alanine methyl ester and the results are shown in Table 1. This technique, to estimate the RMM of unknown molecules has previously been reported, using internal standards of known RMM to construct a correlation graph (Supplementary Materials Figure S2). Whilst this overcomes the problems of viscosity, it does rely upon a similar molecular shape and hydrodynamic radius.

**Figure 5:**
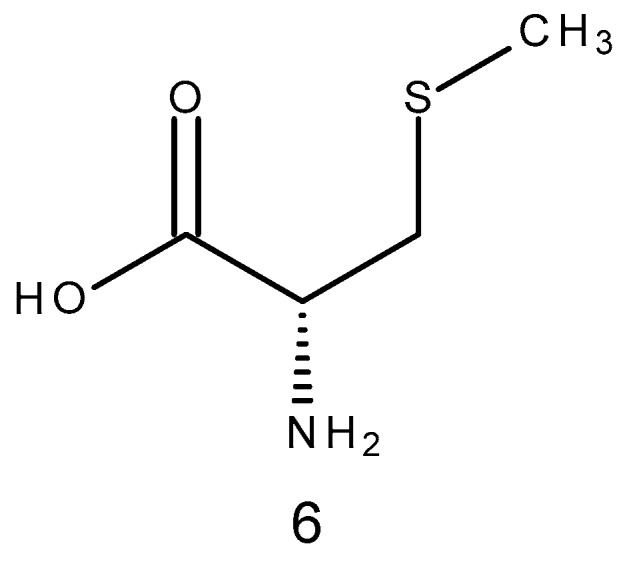
*S*-methyl-*l*-cysteine was used to gain evidence to support the sulfenic acid presence.

Since the evidence pointed towards the postulated cysteine sulfenic acid intermediate **4**, S-methyl-cysteine **6** was prepared using reported methods [10] and similar oxidation with H_2_O_2_ gave rise to NMR and IR data in support of the presence of sulfenic acid and further oxidation to a sulphone (Figure 5) (Supplementary Materials Figure S3).

#### 2.1.2. Synthesis of Cystine Derivatives for Reaction with Sulfite

**Scheme 2:**
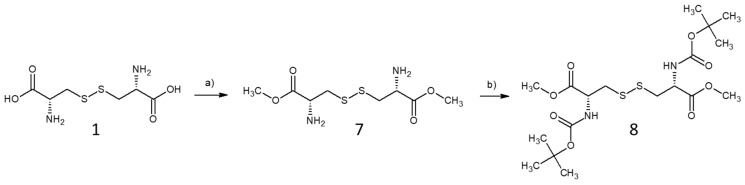
The two steps synthetic procedure to afford the fully protected cystine **8** were (**a**) MeOH, SOCl_2_ from 0 °C to reflux overnight and (**b**) BOC_2_O, Et_3_N, MeOH from 0 °C to r.t overnight.

As a consequence of in situ NMR observations, attempts were made to synthesis a cystine derivative, more soluble in organic solvents to facilitate isolation of the sulfenic acid. The cystine was first methylated to give **7** and then Boc protected as reported in the literature [11] to give **8** (Scheme 2).

#### 2.1.3. Reaction of Cystine Derivatives with Sulfite

**Scheme 3:**
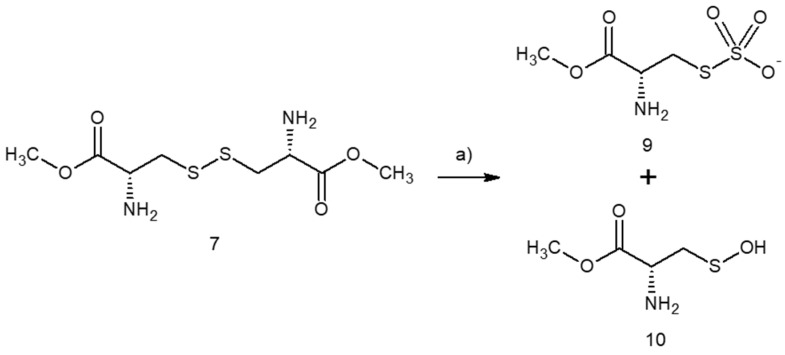
Reaction performed to obtain desired product **9**, where (**a**) Na_2_SO_3_ (2 eq), H_2_O at r.t.

**Figure 6:**
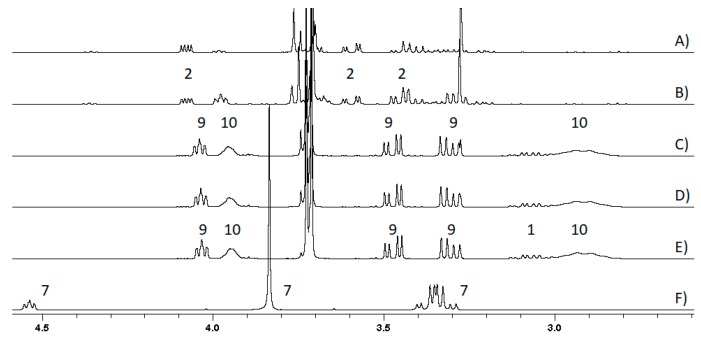
Monitoring of the reaction involving **7** and sodium sulfite in degassed H_2_O at r.t after (**A**) over weekend, (**B**) overnight, (**C**) 4 h, (**D**) 2 h and (**E**) 30 min. (**F**) Cystine methyl ester at t = 0 in D_2_O/H_2_O.

The methyl ester of cystine **7** was reacted with sodium sulfite (see Scheme 3) and monitored by ^1^H-NMR. The spectra seem to evolve as in the cystine:sulfite = 1:2 reaction (Figure 6).

The three sets of signals usually present in the NMR are SSC, sulfenic acid **4** and the residual cystine **1** (Figure 6). This is supported by IR spectra (Supplementary Materials Figure S4) indicating the presence of a S-O vibrational stretch at 1000 cm^−1^.

**Figure 7:**
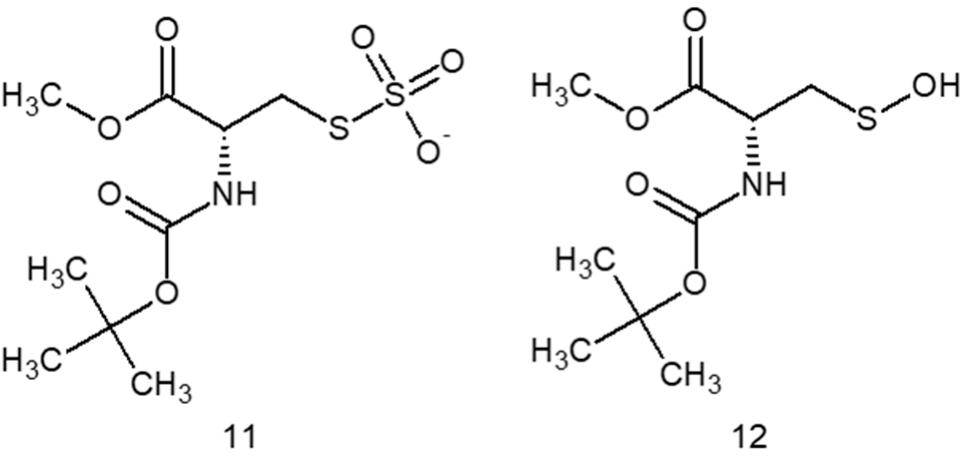
Desired SSC derivatives from compound **8**.

A similar procedure was then followed by using the fully protected cystine, the tert-butoxycarbonyl cystine methyl ester **8**. Due to its higher solubility in organic solvents compared to the cystine methyl ester and cystine itself, the reaction was carried out using a mixture of Dioxane:H_2_O (1:1 and 1:2). The goal of this experiment was to achieve preparation of an organic soluble SSC derivative **11** and evidence the sulfenic acid derivative **12** (Figure 7). Reaction of **8** with sulfite resulted in an intractable mixture, possibly resulting from cyclisation of the formed sulfenic acid (Supplementary Materials Figure S5).

### 2.2. Optimising the Yield of SSC

#### 2.2.1. O_2_ Concentration

O_2_ concentration in distilled water was determined both with and without degassing. The impact of sodium sulfite, cystine and cysteine on O_2_ concentration at different concentrations was also determined (Table 2).

As expected, bubbling nitrogen through distilled water lowers the O_2_ concentration significantly but not completely. The oxygen concentration decreases substantially in the presence of sodium sulfite (for solution at 0.2–0.01 M concentrations), owing to its rapid conversion to sodium sulfate.

Cystine **1** (0.1–0.01 M solutions) is not involved in any redox process, however, cysteine **3** is converted by dissolved oxygen into cystine **1**, which precipitates out of the solution. In the presence of cystine **1**, the sulfite does not appear to undergo oxidation to sulfate as quickly. Based on this, the preferred reaction of sulfite is with cystine to give the SSC and the oxygen level reduction is due to the oxidation of the cysteine anion **13** to cysteine sulfenic acid **4**.

**Scheme 4:**
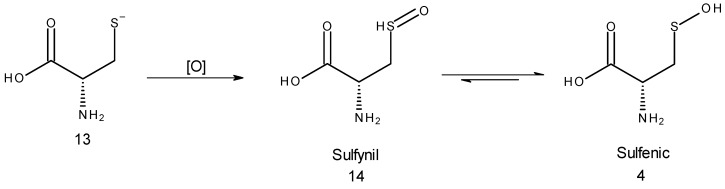
Equilibrium sulfynil-sulfenic acid after oxidation of the thiolate.

The sulfenic acid is in equilibrium between two isomers, and according to the literature, the sulfenic form is slightly more stable (Scheme 4) [10].

**Figure 8:**
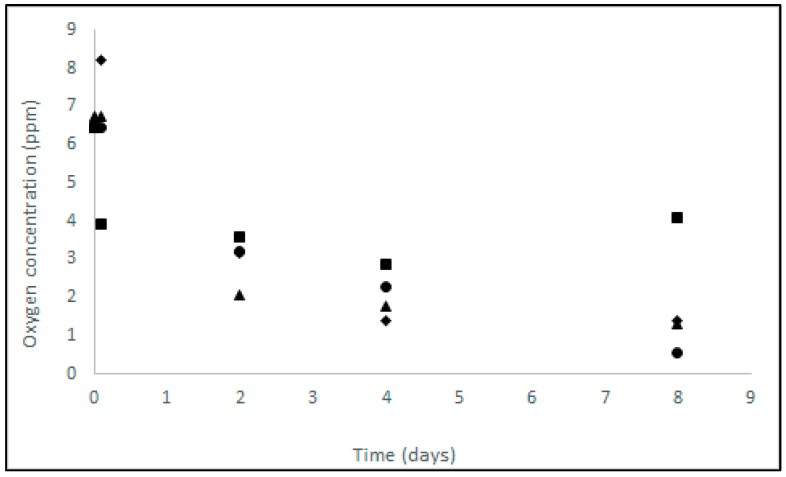
Trend of the oxygen level monitoring over time where ● cystine:sulfite = 1:2, ■ cystine:sulfite = 1:2 at 40 °C, ◆ cystine:sulfite = 1:2 at r.t exposed to the UV light for 6 min before each reading and ▲ cystine:sulfite = 1:2 at r.t exposed to the UV light for 6 min at t = 0.

From the trend visible in Figure 6, it the impact of the UV-light (before each reading (◆) or at t = 0 for all the flasks (▲)) on the reaction composition is negligible in comparison with the trend followed by the reaction mixture left at room temperature (Figure 8). The O_2_ level decreases in all these sets of data, and, as mentioned above, the drop in O_2_ concentration can be associated with the conversion from thiolate to sulfenic acid (Scheme 4). This oxidation seems to be facilitated by the higher oxygen concentration at room temperature and not by the impact on the UV light as the same conversion happens in the presence and absence of the ultraviolet radiation. The reaction mixture involving hot water (40 °C, ■) from t = 0 presents an O_2_ level that could be considered stable for the entire duration of the experiment and due to its lower concentration at 40 °C this conversion is pronounced as at r.t.

#### 2.2.2. pH

**Figure 9:**
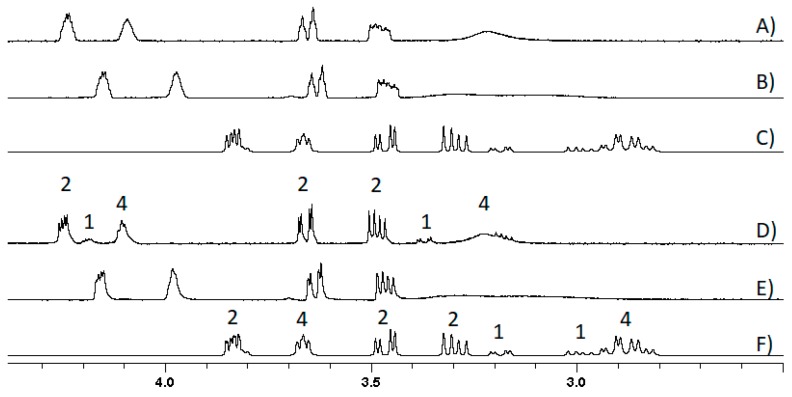
Reaction monitoring after 12 h at (**A**) pH 3, (**B**) pH 5 and (**C**) pH 9 and after 5 min at (**D**) pH 3, (**E**) pH 5 and at (**F**) pH 9 where cystine:sulfite = 1:10 with SO_3_^2−^ = [1 M].

From Figure 9 it is possible to see how the basic pH gives well-resolved NMR signals for the cystine:sulfite aqueous solution, while the acidic environment results in broad and poorly resolved peaks. At pH 4–5 cystine reacts almost completely over a relatively short period of time, howevers at basic pH, there is residual cystine even after 12 h.

Dilution of the mixture was evaluated to determine if a specific pH or reagent concentration, the product formation was favourable.

**Figure 10:**
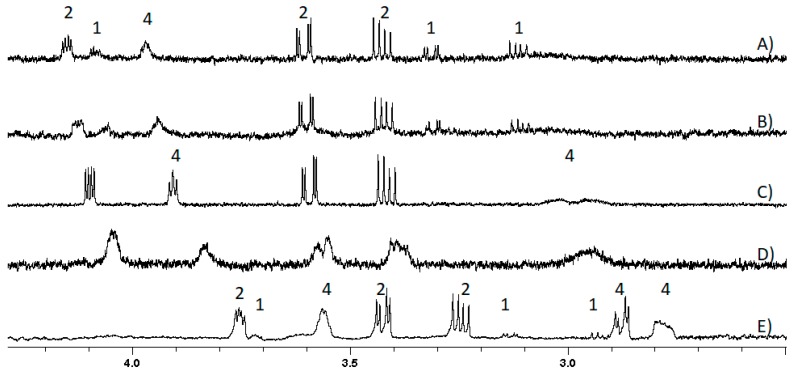
Comparison of ^1^H-NMR spectra when [SO_3_^2−^] = 0.1 M: [cystine] = 0.01 M after 30 min at r.t at (**A**) pH 3, (**B**) pH 4, (**C**) pH 5, (**D**) pH 7 and (**E**) pH 9.

From the stacked spectra (Figure 10), the peak shape for the signal –CH_2_ of the sulfenic acid **4** that varies from 2.8 (pH 9) ppm to 3.1(pH 3) ppm is always very broad. Indeed, it may seem to disappear at acidic pH such as 3–4 but the presence of the other peak at 3.9–4.0 ppm (pH 3–4) belonging to the sulfenic acid is the evidence that this species is still present in the mixture. The mixture at pH 7 gives a very broad spectrum no matter at which time the proton is recorded presumably owing to the zwitterionic nature of the mixture at that pH.

#### 2.2.3. Concentration Ratio Sulfite/Cystine

The reaction mixture was investigated to determine the optimal excess of sodium sulfite required in order to achieve the best reaction time/product yield ratio.

**Figure 11:**
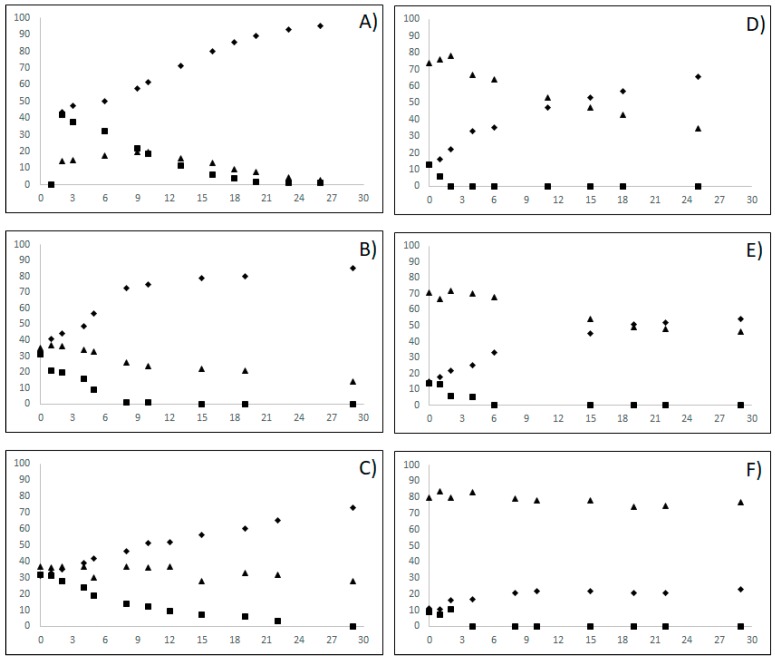
Monitoring of the reaction sulfite:cystine by ^1^H NMR at varying concentrations over 25–29 days. (**A**) 10:1, (**B**) 2:1, (**C**) 1:1, (**D**) 0.5:1, (**E**) 0.25:1 and (**F**) 0.1:1 ratio, where ◆ SSC 2, ▲ cystine 1 and ■ sulfenic acid **4**.

As expected, when low concentrations of sulfite are present (Figure 11), the major component of the reaction is cystine **1**. The relationship between the formation of SSC **2** by conversion of the sulfenic acid **4** into the former can be seen around days 2–8 when the conversion is complete, and both the % of sulfenic acid and product do not change over the next 20 days.

The same trend is visible in the ratio sulfite:cystine = 0.25:1 and 0.5:1 where the cystine is the major component for many days. Despite the sulfite being the limiting reagent, it appears that even after the sulfenic acid **4** is converted completely the % of SSC still increases, and the same can be said for the % of cystine **1**.

Using the ratio 1:1 presents a situation where the three components appear to be in 1:1:1 and the trend is the conversion of sulfenic acid into product until all sulfenic acid **4** is consumed, while the cystine **1** seems to remain at the same percentage.

The sulfite:cystine ratio of 2:1 shows complete consumption of the cystine **1** over time, but sulfenic acid **4** appears to remain. The reaction goes to completion with a sulfite:cystine ratio of 10:1, however from an industrial perspective, this protracted reaction time is unsatisfactory.

#### 2.2.4. UV Irradiation

To determine if both the initial attack of sulfite and/or subsequent reaction with sulfenic acid **4** could be driven by the formation of sulfite radicals [10], samples were irradiated with 254 nm UV light.

**Figure 12:**
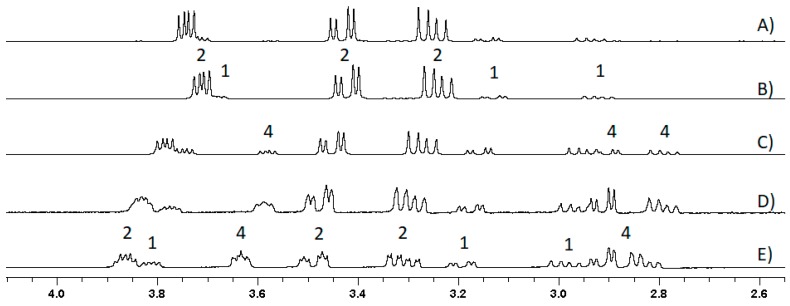
Reaction monitoring after UV light exposure for a 1:2 = cystine:sulfite mixture at pH 9. (**A**) ON with no further exposure to UV light, (**B**) 6 h, (**C**) 5 h, (**D**) 4 h and (**E**) 1 h.

**Figure 13:**
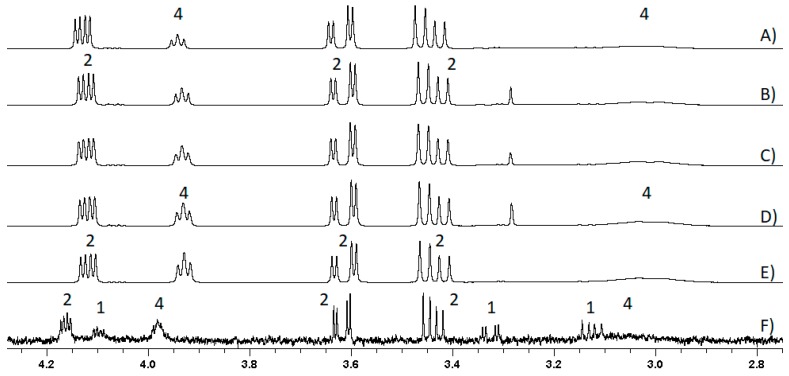
Comparison among the ^1^H-NMR at pH 3 after (**A**) ON with no further exposure to UV light, (**B**) 6 h, (**C**) 5 h, (**D**) 4 h and (**E**) 1 h, (**F**) t = 0.

From Figure 12, it is evident that the UV light at 254 nm influences the transformation of sulfenic acid **4** into SSC, whilst having no effect on the cystine **1** concentration. It is encouraging that in 5 h the % of **4** drops below 5% while the SSC yielded is around 70%. In an attempt to improve the yield of SSC the pH was adjusted to pH 3 before exposure to the mixture to UV light and monitored (Figure 13).

From the reported results it is possible to rationalize the acidic pH is a key condition for the reactivity of cystine, while alkaline conditions combined with UV light exposure are essential to convert the sulfenic acid **4** into the product.

#### 2.2.5. Acid Cycling

**Figure 14:**
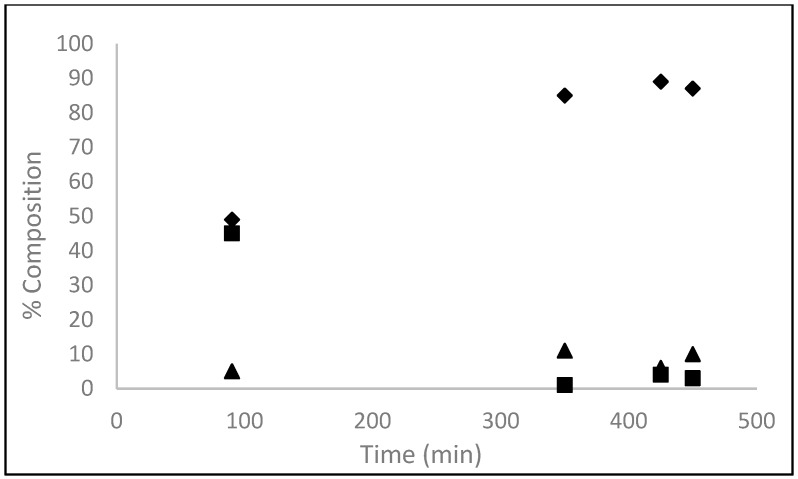
Reaction outcome after acid cycles starting from pH 3 where chronologically the mixture was left at pH 3 for 1 h 30 min, then to pH 9 + UV for 4 h 20 min, then back to pH 3 for 1 h 15 min and then at last back to pH 9 + UV for 25 min, where ◆ SSC 2, ▲ cystine 1 and ■ sulfenic acid **4**.

Based on the pH-sensitive nature of the consumption of **1** and the further reaction of **4**, the idea of acid-base cycling to “eliminate” residual cystine **1** and to convert sulfenic acid **4** into SSC seemed a possible combination to attempt the full conversion of **1** into SSC (Figure 14). The mixture was placed into a petri dish under the UV light when at pH 9 and stirred at r.t away from the UV light when at pH 3.

**Figure 15:**
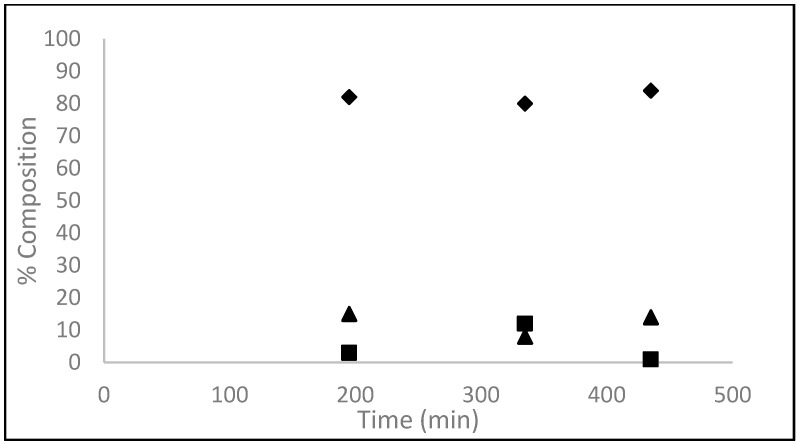
Reaction outcome after acid cycles starting at pH 9 where chronologically the mixture was irradiated with UV light for 3 h 20 min, then at pH 3 for 2 h 20 min and at last back to pH 9 + UV for 1 h, where ◆ SSC 2, ▲ cystine 1 and ■ sulfenic acid **4**.

The acid cycle starting from pH 9 instead of 3 was also carried out, and the results are presented below (Figure 15).

It is clear that a basic environment in combination with the UV light is perfect to drive the reaction of sulfenic acid **4** to SSC **2**. in good yield. In acidic conditions, this conversion is still happening, but not to the same extent as in alkaline conditions. Indeed, at pH 3 the sulfenic acid **4** seem to remain constant for many hours after the initial drop, while the cystine seems to benefit from this environment as the percentage of unreacted starting material is minimal.

From the acid cycling data and the results collected earlier (2.2.4 UV irradiation) the studied reaction needs a combination of acidic pH and UV light exposure under alkaline conditions to progress efficiently towards the formation of SSC **2**.

Low pH is key for the reactivity of cystine, while the basic pH and the exposure to the UV light at this pH is key to the conversion of the sulfenic acid into product, so a combination of both is necessary in order to drive the reaction to completion.

#### 2.2.6. Temperature

**Figure 16:**
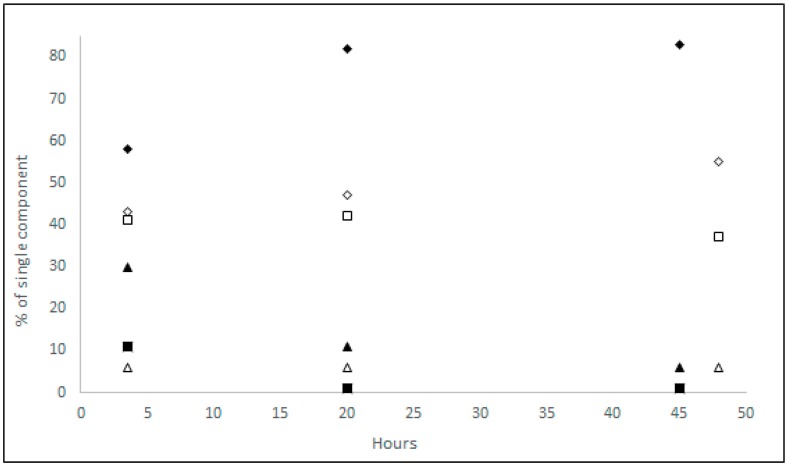
Comparison of temperature effect at pH 4 (△ cystine 1, ☐ sulfenic acid 4 and ◇ SSC 2, and at pH 9 (▲ cystine 1, ■ sulfenic acid 4 and ◆ SSC 2).

To determine the influence of temperature on the yield of SSC **2**, temperature ranges of 60–65 °C and 80–85 °C in the presence and absence of UV light, at various pH were then investigated (Figure 16).

At acidic pH the elevated temperature does not influence the concentration of cystine **1**, the SSC conversion is 50%, which is relatively low. This reinforces the observation that the acidic pH is necessary for the cystine **1** reactivity but not for the conversion of **4** into SSC.

At basic pH, SSC is obtained in much higher yield and **4** drops to a low level, but it takes longer in the absence of UV light (20 h instead of 3–5 h). The conversion reached at the end of the experiment is also not very satisfactory, implying that the temperature has a limited effect when compared with the direct exposure to UV light.

Further elevation of the temperature to 85 °C supports the observation that UV has a more pronounced effect on overall yield than temperature (Supplementary Materials Figures S6 and S7).

## 3. Discussion

### 3.1. Mechanism

**Scheme 5:**
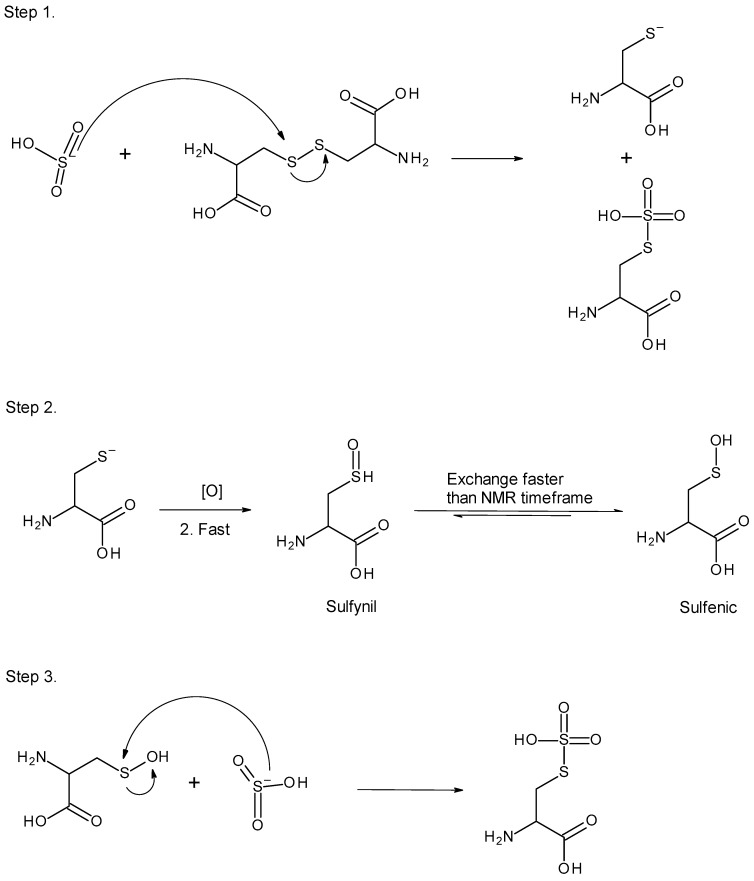
Proposed reaction mechanism for the ionic reaction (Step 3 is favored under high pH).

The mechanism for the reaction of sulfite with cystine has previously been reported as producing cysteine and SSC [7], however, the extensive NMR studies undertaken in this paper, indicate that there exists a rapid oxidation step converting cysteine into sulfenic acid. This then reacts with excess sulfite to provide SSC as the exclusive product. We have shown the presence of the sulfenic acid by NMR and postulate that depending on the pH, it is in equilibrium between its sulfinyl and sulfenic forms, resulting in broadening of the observed NMR signals. We also are able to discount the presence of cysteine alone as a by-product owing to the substantial chemical shift difference observed and also the lack of the appropriate diffusion coefficient. Whilst attempts to isolate the sulfenic acid and characterize it using MS were unsuccessful, active oxidation of cysteine and reactions of synthesized cystine derivative could be used to evidence the formation of sulfenic acid using both IR and NMR. Previous research [3] has indicated that SSC can only be generated via the in situ action of an oxidizing metal compound. Herein, we report for the first time, evidence for the in situ formation of sulfenic acid from cystine via a mechanism (Scheme 5), which only requires dissolved oxygen. This may provide resolution to the contrary conclusions reported by Bryant and Weitzmann [12,13].

### 3.2. SSC Generation

The formation of SSC in solution from the reaction of sulfite with cysteine appears largely independent of temperature, however, the reaction rates for the initial attack of the sulfite ion and subsequent sulfenic acid reaction would appear to be heavily pH dependent. The acid cycling studies suggest that yields of SSC can be increased by varying the pH to increase the velocity of these individual reactions (Scheme 5). It is also important to specify that the pH of the reaction below does not change from the initial value (pH 8–9) if left running at r.t and the reaction is in fact very slow to proceed.

It is noteworthy that UV irradiation has a significant effect on the conversion of sulfenic acid into the SSC and this further suggests an additional mechanism, potentially using a sulfite radical, which can be formed under these conditions. Whilst ESR studies with a DMPO spin trap were unable to verify the presence of any stable radical species, it warrants further study.

Herein, we provide, for the first time, comprehensive reaction conditions to facilitate the highest yield of SSC and in doing so have elucidated a potential, previously unreported mechanism, which adds to our understanding of this reaction for the first time in almost 90 years.

## 4. Materials and Methods

### 4.1. General Experimental

All NMR experiments were carried out on a Bruker Avance III 600 MHz, Bruker Biospin GmbH, (Rheinstetten, Germany) with TXI probe and a temperature control unit was used for all 1D and 2D experiments. The 5-mm single use NMR tubes were purchased from GPE Scientific, Leighton Buzzard, UK) (product code 512-7). The spectra were recorded using Deuterium oxide (99.90% D) purchased from Fluorochem (Glossop, UK). l-cystine (≥ 99.7% by TLC) and sodium sulfite (≥98%) were purchased from Sigma-Aldrich, Haverhill, UK and used without further purification. Distilled H_2_O was generating using an ELGA water still and degassed using N_2_ gas (BOC, 99.99%)

#### 4.1.1. O_2_

O_2_ concentration was measured using a Hanna HI9146 (Hanna Instruments Ltd., Leighton Buzzard, UK) dissolved oxygen and temperature meter purchased from Hanna Instruments and calibrated according to the instructions available on the website https://www.presens.de/support-services/faqs/question/.

#### 4.1.2. pH

Solution pH was measured using the pH strips indicator MColorpHast^®^ product available at Merck Millipore (Watford, UK). The pH was adjusted using a 1 M NaOH or 1 M HCl solution prepared respectively from NaOH pellet (99%) and HCl (36.5%) purchased from VWR Chemicals. In one particular experiment, the reaction mixture was buffered to pH 9 using the ATX Tris-buffer (ready to use solution) purchased from Sigma-Aldrich.

#### 4.1.3. Concentrations

With regards to the oxidation of cysteine by H_2_O_2_ as shown in Figure 3, [cysteine] = 4 M. The DOSY experiment reported in Figure 4 was record on a mixture cystine:sulfite = 1:10 where [SO_3_^2−^] = 0.1 M at pH 9. The same sulfite concentration was involved in the reaction mixture under investigation in Figure 5. All the oxygen monitoring experiments (Figure 6) were performed using a cystine:sulfite = 1:2 with [SO_3_^2−^] = 0.2 M. Concentrations were calculated to a maximum possible cystine concentration in solution at [cystine] = 0.01 M in all the experiments reported in Figure 9. For the experiment reported in Figure 10 and Figure 11 a cystine:sulfite = 1:2 mixture having [SO_3_^2−^] = 0.2 M was involved. The same concentration for sulfite (0.2 M) and cystine (0.1 M) was used for the acid cycles experiments (Figure 15 and Figure 16), and for the experiments to evaluate the effect of pH and temperature in Figure 14.

#### 4.1.4. UV

UV experiments were carried out using a Uvitec UV-lamp (LF-206.LS, Cleaver Scientific, Rugby, UK) irradiating at 254 and 365 nm and placed at a distance of 5 cm from the sample.

#### 4.1.5. Acid Cycling

The reaction mixture cystine:sulfite = 1:2 in H_2_O is normally at pH 8–9, so in order to perform the base-acid cycle the original mixture is placed underneath the UV lamp and the area kept in the dark for the desired time. The reaction mixture is then removed from the UV lamp and acidified to the desired pH using 1 M HCl and kept on a flat surface, in air for the specified time. In order to perform the acid-basic cycle, the reaction was taken to pH 3 using 1 M HCl, immediately after the reagents were mixed, and the reaction vessel left on a flat surface in air for the indicated time. The same mixture was then buffered back to pH 8–9 using 1 M NaOH while stirring. When at the correct pH, the reaction vessel was placed underneath the UV lamp described in Section 4.1.4 for the indicated time. The process was then repeated according to the desired number of cycles.

#### 4.1.6. Temperature

The temperatures indicated were taken using a mercury thermometer placed inside the round-bottom flasks. In the case of the O_2_ monitoring experiments (Figure 9), the flasks were stored inside a Gallenkamp vacuum oven where the temperature was set to 40 °C and a mercury thermometer was placed inside to measure the correct temperature, which resulted in the range 42–43 °C.

## Figures and Tables

**Table 1:** The values and the calculation are shown based on the known MW of cystine, l-Ala-Me ester and sulfocysteine. MW obtained from the experimental data. ^α^) theoretical MW of the “Intermediate” and ^β^) expected MW used to re-calculate the logMW.

Entry	Diff.coeff	MW	logMW	MW	logMW
Cystine	9.646	240	2.380	240	2.380
SSC	9.577	200	2.301	200	2.301
l-Ala-Me ester	9.331	102	2.009	102	2.009
Intermediate	9.452	142.9 ^α^	2.155	137 ^β^	2.137

**Table 2:** Oxygen concentration in ppm over time for various solutions.

	Conc.	Blank	<5′	4 h	20 h	2 d	3 d
H_2_O-distilled	n/a	7.65	n/a	n/a	n/a	n/a	n/a
H_2_O-degassed (N_2_-30/60 min)	n/a	2.50	n/a	n/a	n/a	n/a	n/a
Na_2_SO_3_	0.01 M	7.59	4.92	0.00	n/a	n/a	n/a
Cystine	0.01 M	7.29	7.36	7.38			
Cysteine	0.1 M	7.04	7.13	/	7.12	4.95	1.65
Cystine:Sulfite = 1:2	0.2 M	7.14	7.55	/	3.18		

## Supplementary Materials

The following are available online at https://www.mdpi.com/1420-3049/24/13/2377/s1, S1: NMR spectra showing cysteine at pH 8-9, S2: Linearity of diffusion correlation with MW, S3: NMR and FT-IR data of S-methyl-L-cysteine, S4: NMR and FT-IR in support of the presence of sulfenic acid and further oxidation to a sulphone ^1^H-NMR, S5: Reaction of 8 with sulfite resulted in an intractable mixture, possibly resulting from cyclisation of the formed sulfenic acid, S6: Combined Temperature and UV effects with varying pH.

## References

[1] Hannah-Shmounia F., MacNeil L. (2019). Severe cystic degeneration and intractable seizures in a newborn with molybdenum cofactor deficiency type B. Mol. Genet. Metab. Rep..

[2] Segel I.H., Johnson M.J. (1963). Synthesis and Characterization of Sodium Cysteine-S-sulfate Monohydrate. Anal. Biochem..

[3] Hohowitz N.H. (1950). Biochemical Geenetics of Neurospora. Adv. Genet..

[4] Hockenhull D.J.D. (1949). The sulphur metabolism of mould fungi: The use of “biochemical mutant” strains of Aspergillus nidulans in elucidating the biosynthesis of cystine. Biochim. Biophys. Acta.

[5] Nakamura T., Sato R. (1960). Cysteine-*S*-sulphonate as an Intermediate in Microbial Synthesis of Cysteine. Nature.

[6] Kassell B., Brand E. (1938). Phosphotungstic acid related compounds with cysteine, ascorbic acid, and the photometric determination of cystine. J. Biol. Chem..

[7] Clarke H.T. (1932). The action of sulfite upon cystine. J. Biol. Chem..

[8] Goto K., Holler M., Okazaki R. (1997). Synthesis, Structure, and Reactions of a Sulfenic Acid Bearing a Novel Bowl-Type Substituent: The First Synthesis of a Stable Sulfenic Acid by Direct Oxidation of a Thiol. J. Am. Chem. Soc..

[9] Le Gresley A., Simpson E., Sinclair A., Williams N., Burnett G., Bradshaw D., Lucas R. (2015). The application of high resolution diffusion NMR for the characterisation and quantification of small molecules in saliva/dentifrice slurries. Anal. Methods.

[10] Amtul Z., Kausar N., Follmer C., Rozmahel R.F., Atta-Ur-Rahman, Kazmi S.A., Shekhani M.S., Eriksen J.L., Khan K.M., Choudhary M.I. (2006). Cysteine based novel noncompetitive inhibitors of urease (s)—Distinctive inhibition susceptibility of microbial and plant ureases. Bioorg. Med. Chem..

[11] Busnel O., Carreaux F., Carboni B., Pethe S., Vadon-Le Goff S., Mansuy D., Boucher J.L. (2005). Synthesis and evaluation of new ω-borono-α-amino acids as rat liver arginase inhibitors. Bioorg. Med. Chem..

[12] Man M., Bryant R.G. (1974). Reactions of thiosulfate and sulfite ions with DTNB: Interference in sulfhydryl group analysis. Anal. Biochem..

[13] Weitzmann P.D.J. (1975). A critical reexamination of the reaction of sulfite with DTNB. Anal. Biochem..

