# Supplementary material for: New Insights into the Cystine-Sulfite Reaction
Source: Molecules. 2019 Jun 27;24(13):2377. doi: 10.3390/molecules24132377 (PMC6650951; doi:10.3390/molecules24132377)
Supplement: Supplementary file 1 [file molecules-24-02377-s001.pdf]

New insights into the cystine-sulfite reaction

Matteo Zecchini[1], Robert Lucas [2] and Adam Le Gresley [1,*]

[1] Kingston University, Surrey, KT1 2EE, UK; m.zecchini@kingston.ac.uk

[2] GlaxoSmithKline, Consumer Healthcare, Weybridge, UK; Robert.a.lucas@gsk.com

* Correspondence: a.legresley@kingston.ac.uk; Tel.: +44-(0)208-4177432

Supporting information

S1 NMR spectrum showing cysteine at pH 8-9

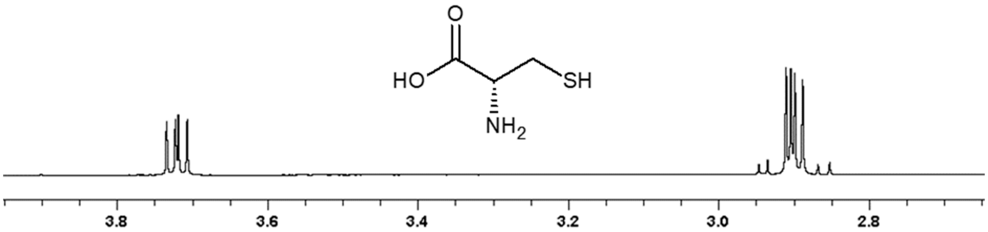

Proton NMR of Cysteine in D_2O at pH 8-9.

S2 Linearity of diffusion correlation with MW

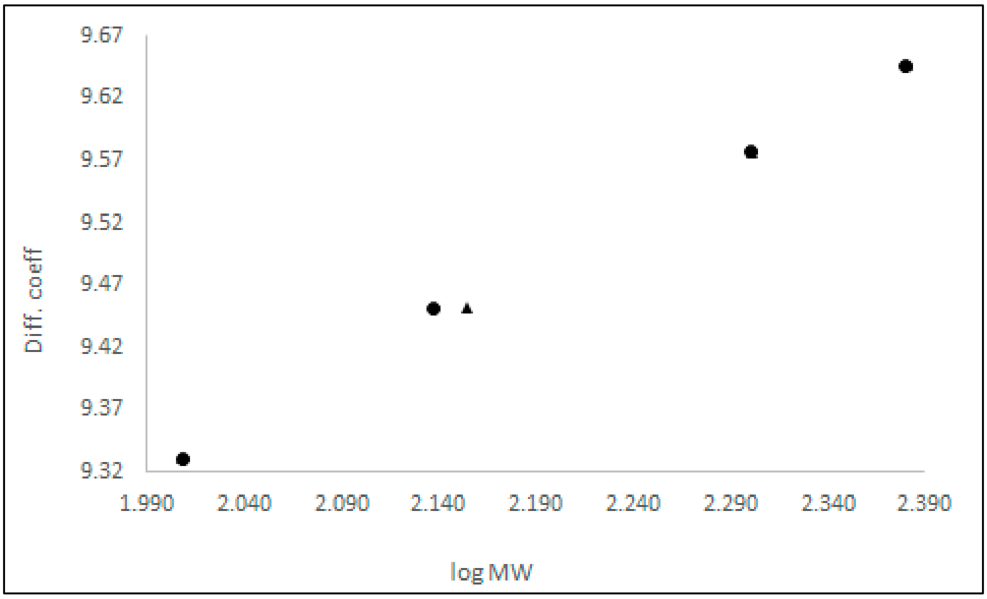

Theoretical (●) and experimental (▲) correlation of LogMW vs LogD show good linearity, where in details ●: y=0.8477x + 7.6271, R^2=0.9999 and ▲: y=0.8353x + 7.6582, R^2=0.9981.

S3 NMR and FT-IR data of S-methyl-L-cysteine

^{1}H-NMR.

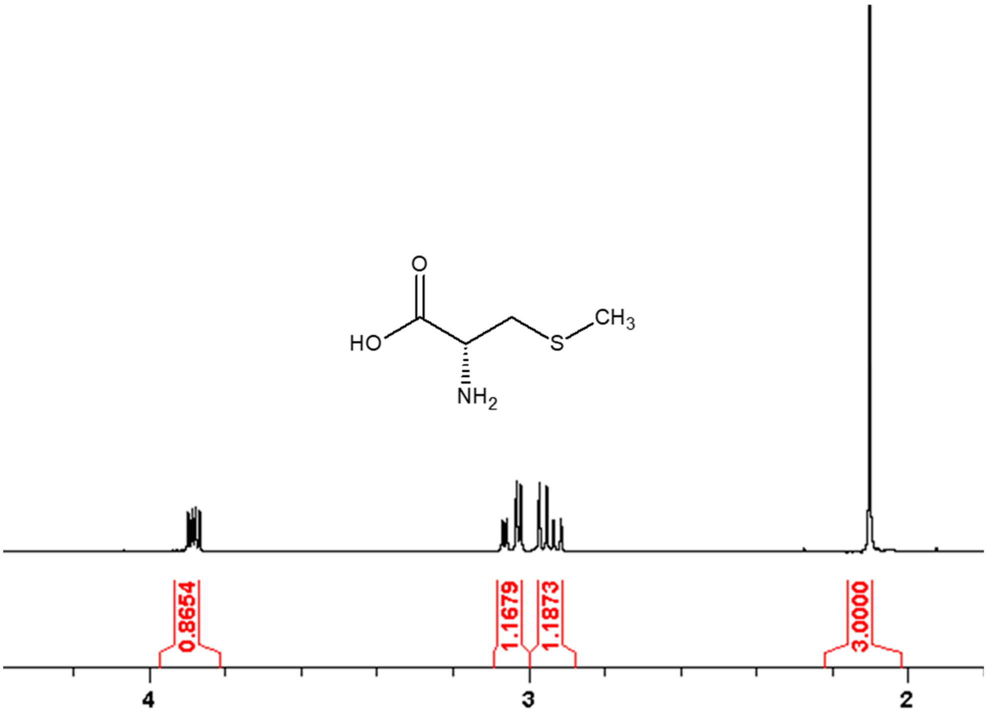

Proton NMR of S-methyl-L-cysteine in D2O.

FT-IR.

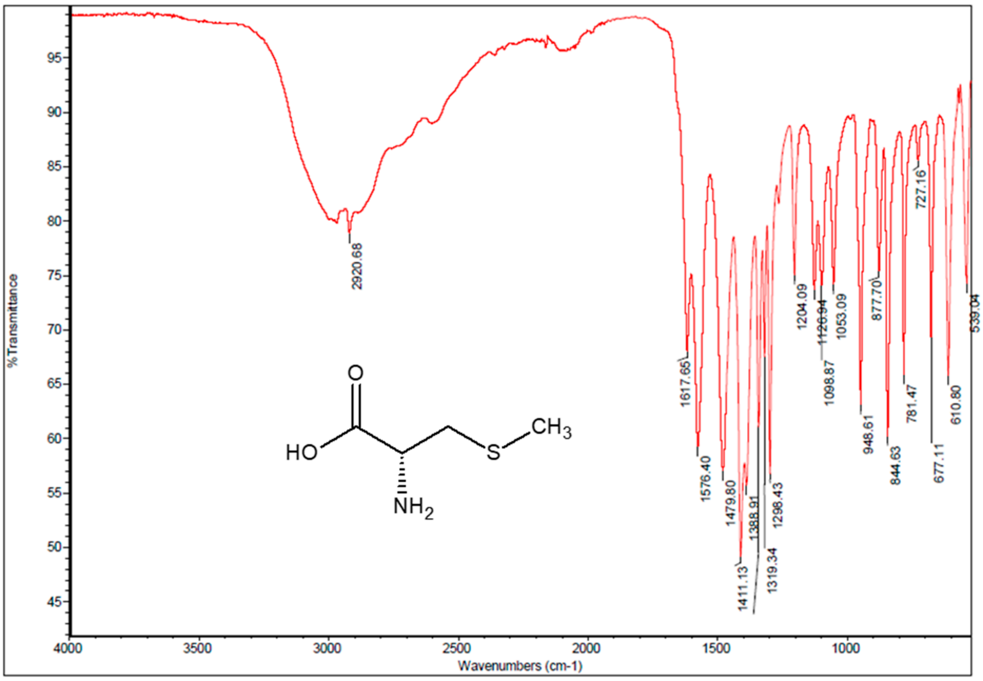

FT-IR of S-methyl-L-cysteine obtained as a white solid.

S4 NMR and FT-IR in support of the presence of sulfenic acid and further oxidation to a sulphone

^{1}H-NMR.

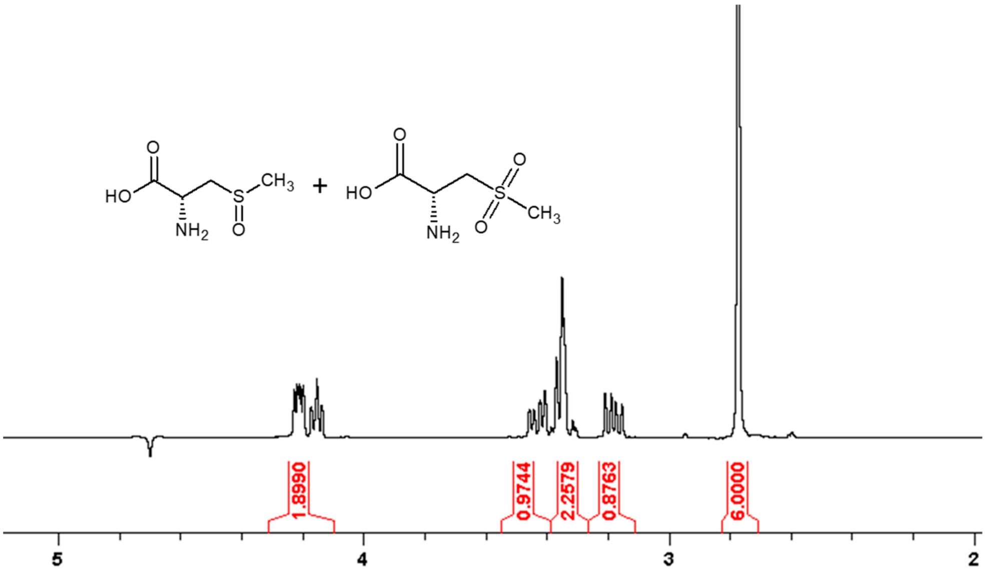

Proton NMR of the mixture of Sulfoxide and Sulfone given by the oxidation of the S-Me-L-Cysteine.

FT-IR.

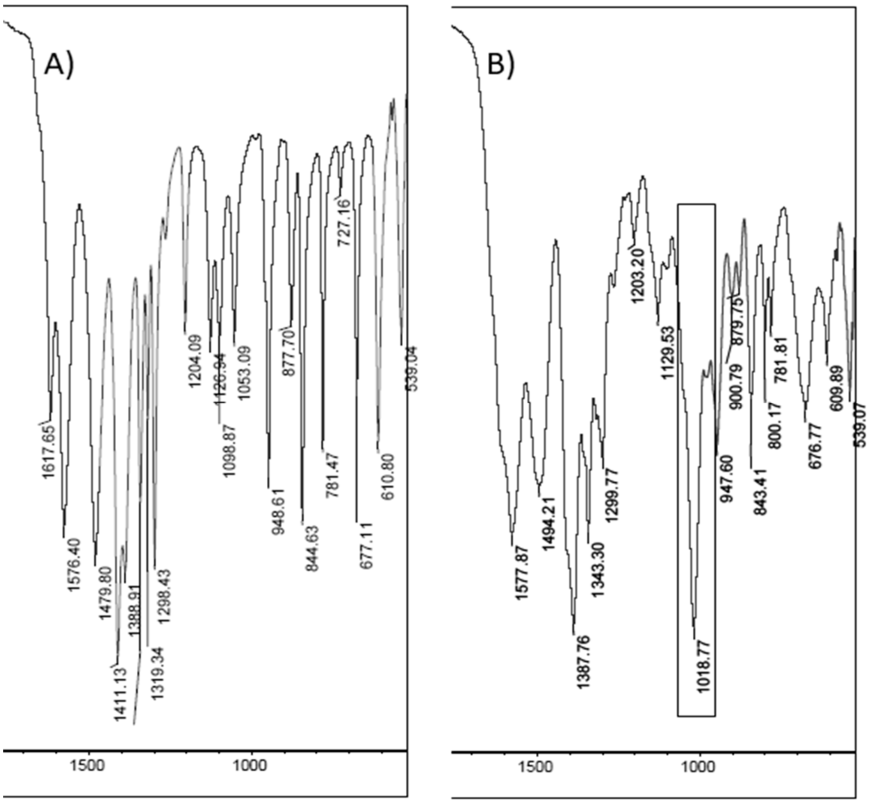

FT-IR spectra of reactiom. The intense stretching around 1000 cm-1 (spectrum B) could be the S-O bond not present in the SM (spectrum A).

S5 Reaction of **8** with sulfite resulted in an intractable mixture, possibly resulting from cyclisation of the formed sulfenic acid (Supporting Information S5)

^{1}H-NMR of **8**.

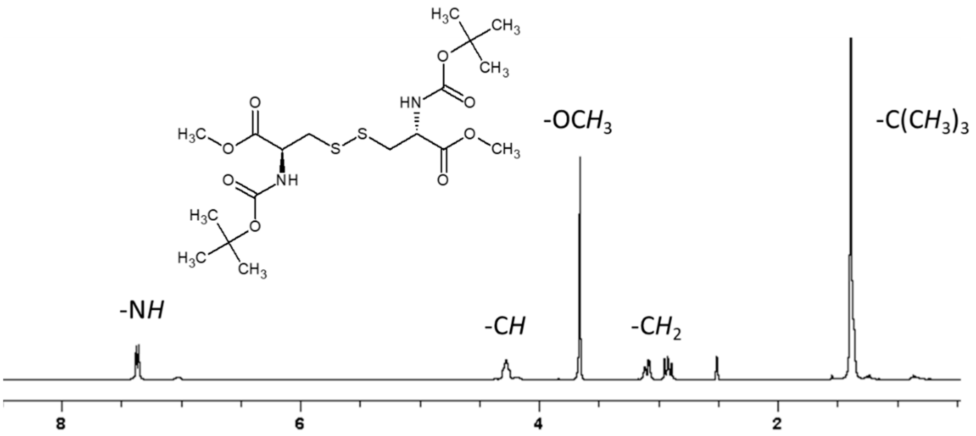

Proton NMR for starting material **8** in DMSO-d6.

^{1}H-NMR for the crude reaction mixture.

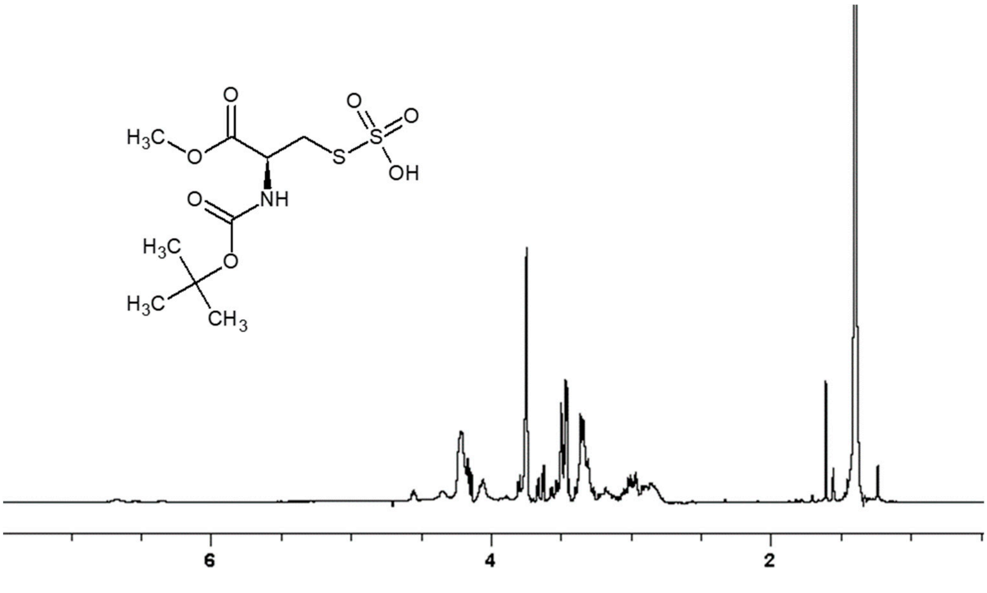

Proton NMR for an aliquot of the reaction mixture dried under nitrogen (80°C-12h). Spectrum recorded in H_2O/D_2O. The desired compound was not identified in the crude mixture.

S6 Combined Temperature and UV effects with varying pH

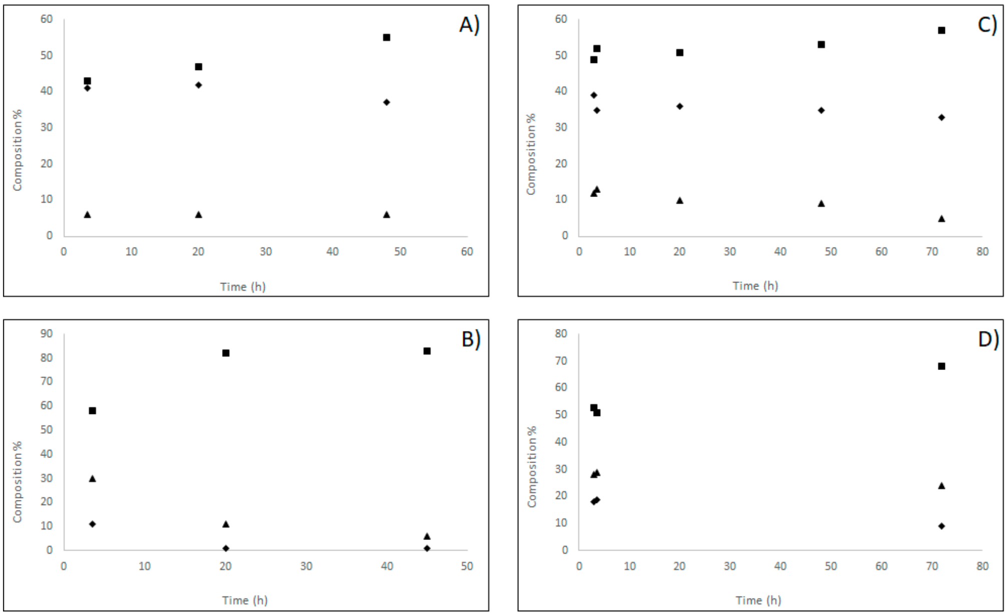

Comparison between the effect of temperature (65°C) at A) pH4 and B) pH9 and effect of UV light (30s-254nm) and temperature (65°C) at C) pH4 and D) pH9.

Table 1. Variation in the reaction mixture percentage composition after UV exposure at high temperature.

pH9-85°C	Time (h)	2 (%)	4 (%)	1 (%)
	2	37	34	28
	2.5	42	31	23
+ 10s UV-254nm				
	3	42	31	23
	20	53	26	18
	48	55	19	18
+ 30s UV-254nm				
	3	39	31	25
	20	47	28	20
	48	57	20	19
